# Selected HIV-1 Env Trimeric Formulations Act as Potent Immunogens in a Rabbit Vaccination Model

**DOI:** 10.1371/journal.pone.0074552

**Published:** 2013-09-02

**Authors:** Leo Heyndrickx, Guillaume Stewart-Jones, Marianne Jansson, Hanneke Schuitemaker, Emma Bowles, Luigi Buonaguro, Berit Grevstad, Lasse Vinner, Katleen Vereecken, Joe Parker, Meghna Ramaswamy, Priscilla Biswas, Guido Vanham, Gabriella Scarlatti, Anders Fomsgaard, on behalf of the NGIN Consortium

**Affiliations:** 1 Biomedical Department, Virology Unit, Institute of Tropical Medicine, Antwerp, Belgium; 2 Human Immunology Unit, Weatherall Institute of Molecular Medicine, University of Oxford, Oxford, United Kingdom; 3 Department of Laboratory Medicine, Lund University, Lund, Sweden; 4 Department of Experimental Immunology, Academic Medical Center at the University of Amsterdam, Amsterdam, The Netherlands; 5 Molecular Biology and Viral Oncogenesis Unit, Istituto Nazionale Tumori, Naples, Italy; 6 Statens Serum Institut, Copenhagen, Denmark; 7 National Institute for Biological Standards and Control, Hertfordshire, United Kingdom; 8 Viral Evolution and Transmission Unit, Division of Immunology, Transplantation and Infectious Diseases, San Raffaele Scientific Institute, Milan, Italy; 9 Biomedical Department, University of Antwerp, Antwerp, Belgium; 10 Clinical Institute, University of Southern Denmark, Odense, Denmark; New York University, United States of America

## Abstract

**Background:**

Ten to 30% of HIV-1 infected subjects develop broadly neutralizing antibodies (bNAbs) during chronic infection. We hypothesized that immunizing rabbits with viral envelope glycoproteins (Envs) from these patients may induce bNAbs, when formulated as a trimeric protein and in the presence of an adjuvant.

**Methods:**

Based on *in vitro* neutralizing activity in serum, patients with bNAbs were selected for cloning of their HIV-1 Env. Seven stable soluble trimeric gp140 proteins were generated from sequences derived from four adults and two children infected with either clade A or B HIV-1. From one of the clade A Envs both the monomeric and trimeric Env were produced for comparison. Rabbits were immunized with soluble gp120 or trimeric gp140 proteins in combination with the adjuvant dimethyl dioctadecyl ammonium/trehalose dibehenate (CAF01). Env binding in rabbit immune serum was determined using ELISAs based on gp120-IIIB protein. Neutralizing activity of IgG purified from rabbit immune sera was measured with the pseudovirus-TZMbl assay and a PBMC-based neutralization assay for selected experiments.

**Results:**

It was initially established that gp140 trimers induce better antibody responses over gp120 monomers and that the adjuvant CAF01 was necessary for such strong responses. Gp140 trimers, based on HIV-1 variants from patients with bNAbs, were able to elicit both gp120_IIIB_ specific IgG and NAbs to Tier 1 viruses of different subtypes. Potency of NAbs closely correlated with titers, and an gp120-binding IgG titer above a threshold of 100,000 was predictive of neutralization capability. Finally, peptide inhibition experiments showed that a large fraction of the neutralizing IgG was directed against the gp120 V3 region.

**Conclusions:**

Our results indicate that the strategy of reverse immunology based on selected Env sequences is promising when immunogens are delivered as stabilized trimers in CAF01 adjuvant and that the rabbit is a valuable model for HIV vaccine studies.

## Introduction

Broadly neutralizing antibody (bNAb) responses are considered to be an important component of preventive human immunodeficiency virus type 1 (HIV-1) vaccines. Passive transfer of bNAbs can protect non-human primates against infection with SHIV (i.e. simian immune deficiency virus with an HIV-1 envelope) [1], [2], [3], [4], [5], [6], [7]. Many efforts to design immunogens for the induction of antibodies against neutralizing determinants of the HIV-1 envelope glycoprotein gp120/gp41 complex (Env), however, have met with limited success so far. Hypervariability of the *env* gene, extensive glycosylation of the Env protein and instability of the native trimer is thought to contribute to the difficulty to elicit Env-specific bNAbs [8], [9].

Interestingly, about 10% – 30% of HIV-1-infected individuals develop bNAbs about 2-3 years after infection, suggesting that these Abs result from an elaborate selection and maturation process driven by continuous viral evolution [10], [11], [12]. Not surprisingly therefore, all broadly neutralizing monoclonal Abs isolated so far have been found to carry elevated frequency (15% – 44%) of somatic mutations [13], [14]. Intensive efforts have focused on identifying and characterizing target structures of such naturally occurring bNAbs [15], [16], [17], [18].

Here we hypothesized that viral variants from patients who display bNAb responses, may harbor specific Env structures that could elicit bNAbs in animals if formulated into an appropriate vaccine immunogen. We therefore focused on Env immunogens from recently infected subjects, who later developed bNAbs, as well as on Envs from chronically infected individuals with contemporaneous bNAbs. The capacity of these Envs to elicit bNAbs was studied using an established rabbit vaccination model [19]. The induced antibody response was assessed for its capacity to bind to gp120 and to neutralize HIV strains *in vitro*, with emphasis on kinetics, potency and breadth.

## Materials and Methods

### Selection of primary HIV-1 Envs for immunogens

Envs included in the study were derived from patients infected with either clade A or B HIV-1 who showed particularly broad neutralizing activity in their serum in a variety of *in vitro* assays [20], [21], [22], [23] (and data not shown). From a male long term survivor, infected with a clade A virus by mother to child transmission, both the earliest available isolate, i.e. 11 years after infection (ITM1_4) and the predicted ‘ancestral’ Env (ITM_anc), as based on 178 full-length *env* sequences spanning 11 years, were used. Another clade A Env (94UG018, herein referred to as UG_A) was derived from an asymptomatic pregnant woman [24], [25]. In addition, two subtype B Envs were derived from viruses isolated during the first months of life from neonatally infected children, i.e. isolate 306-9 (herein referred to as CHILD_1) and isolate 136-3 (herein referred to as CHILD_2) [26], [27]. Finally, two subtype B Envs were derived from adult male patients 2 to 4 years after MSM transmission (ACS19642 and ACS19554). In addition the reference Bx08 subtype B Env was used in a pilot experiment.

### Expression and purification of recombinant monomeric and trimeric gp120 and gp140

All HIV-1 Env gp120 monomers and gp140 trimers were produced following transient transfection of HEK293T cells cultured in high glucose DMEM (Sigma) supplemented with 10% Fetal Calf Serum (FCS, Sigma) and Penicillin-Streptomycin solution (Sigma). Individual protein expression constructs were generated by cloning each DNA *env* into a pLEXm vector (kind gift from Radu Ariescu at the Division of Structural Biology, WTCHG, Oxford) containing an N-terminal His tag to allow purification. Two mg plasmid DNA was first incubated with 3.6 mg polyethylenimine (PEI) in media without FCS for 30 minutes to allow complex formation. These DNA/PEI complexes were added to 293T cells and the cultures were filled up to 500 ml with DMEM containing 2% FCS and incubated in multilayer Cell Bind Hyperflasks (Corning). Supernatants were collected after 48 hours and fresh media, containing 10% FCS was added to the cells for another 48 hours at which time the media was exchanged again. The supernatant, pooled from all time points of each separate transfection, was centrifuged at 7000 x g for 4 hours to remove cell debris, and passed through a 0.22 µm filter (Millipore) using a vacuum pump. After adjusting to pH 8 using 1 M Tris HCl (Sigma), the supernatant was passed over a cobalt chloride metal-affinity column made of Talon superflow resin (Clontech) to specifically bind the his-tagged Env protein. The resin was washed with 2 column volumes of 0.015 M Tris Buffered Saline (Sigma) and the protein eluted using 250 mM imidazole. The eluted fraction, containing the gp140 protein, was concentrated and separated by gel filtration chromatography using a Superdex200 26/60 size-exclusion column (GE Healthcare). Individual fractions were run on 4–12% Bis-Tris gels (Invitrogen) which were then stained with Coomassie Blue (Sigma). Fractions corresponding to the trimer were identified and further purified by passing over a GNA-lectin resin (Vectorlabs) that specifically binds glycoprotein. Protein was passed through a further size exclusion chromatography (SEC) fractionation and fractions were again run on 4–12% Bis-Tris gels (Invitrogen) to allow identification of pure gp140 or gp120 protein. Fractions were pooled and concentrated prior to immunization.

### Rabbit immunizations

All animal experiments were performed in accordance with the Animal Experimentation Act of Denmark and European Convention ETS 123 (Protection of Vertebrate Animals used for experimental and other scientific purposes). Ten week-old New Zealand White nulliparous female rabbits (Charles River Laboratories) were housed at the Animal facility at Statens Serum Institut (SSI), Copenhagen, Denmark with an acclimatization period of at least 10 days. Thus all experimental results were obtained in animals aged between 12 and 28 weeks. Groups of 4 rabbits were each immunized subcutaneously (s.c.) with Env gp140 trimers (100 µg/dose) at weeks 0, 2, 4 and 8 in the presence (or absence) of cationic adjuvant formulation number 1 (CAF01) [28], [29] (total 400 µl). The two adjuvant components Dimethyldioctadecylammonium bromide (DDA) and Trehalose-Dibehenate (TDB) of CAF01 were synthetically manufactured by Avanti Polar Lipids, AL, USA and produced GMP at SSI by the lipid film hydration method, as previously described [29], [30]. The dose of 100 µg trimer was chosen based on a pilot experiment comparing different immunization doses and was used throughout this study for all trimeric and monomeric Env immunizations except when otherwise stated.

Ear bleeding was performed before each immunization and also 4 and 6 weeks after the last immunization. Animals were sacrificed 6 weeks after the last immunization (week 14) and all sera stored at –20°C.

### Anti-Env antibody Enzyme-linked immunosorbent assay (ELISA)

For measurement of specific anti-gp120 IgG antibodies 96-well Maxisorp plates (Nunc) were coated overnight at 4°C with 2 µg/ml rgp120 IIIB (Fitzgerald industries Int., Acton, Maryland, USA) in carbonate buffer, pH 9.6. Plates were blocked for 1 hour at room temperature in PBS containing 1% Triton-X-100, 1% BSA and 10% FCS, pH 7.2 and washed 3×1 minute with washing buffer (PBS, 0.1% Triton-X-100), before incubation overnight with rabbit sera diluted in dilution buffer (PBS, 1% Triton-X-100 and 1% BSA, pH 7.2). The plates were washed 5 x with washing buffer for 1 minute and IgG antibodies detected by HRP-conjugated mouse anti-rabbit IgG (A1949, Sigma) diluted 1∶200 in the blocking buffer for 1 hour at room temperature. After 5×1 minute washes with washing buffer, the reaction was developed by TMB ready-to-use substrate (Kem-En-Tec Diagnostics, Copenhagen, Denmark) and the colorimetric reaction stopped with 0.2 M H_2_SO_4_ after 30 minutes. Absorbance was read at 540 nm with a 620 nm reference.

Titers were defined as the reciprocal dilution yielding an absorbance value greater than the OD of twice the background OD (wells containing blocking buffer only). The positive standard consisted of pooled rabbit antisera with known high titers of gp120-specific IgG as tested previously [19].

### Neutralization assays

IgG was purified from heat inactivated (1 hour 56°C) serum using Protein G HP SpinTrap columns (GE Healthcare) according to the manufacturer’s instructions. Eluted IgG fractions were quantified spectrophotometrically (Nanodrop). The purified IgG from different sera were assayed in pseudovirus neutralization assays using TZMbl cells conducted in triplicate as described previously [31], [32], www.europrise.org/neutnet_sops.html; SOP2. Briefly, four 2-fold IgG dilutions starting at a final concentration of 250 µg/ml were mixed with pseudovirus and incubated for 1 hour at 37°C in a 96 well plate before adding TZMbl cells (10^4^/well). Final IgG concentrations were calculated from virus-inhibitory reagent mixtures, before addition of cells. Infection levels were determined after 48 hour by measuring firefly luciferase activity and the percentage of calculated neutralization was relative to the virus control (no IgG added). TriMab, a mix of 3 mAbs (b12, 2G12 and 2F5) (obtained from Centre for AIDS Reagents, NIBSC, UK), was used in every neutralization experiment as a strongly neutralizing control IgG. In selected experiments neutralizing activity of rabbit IgG was assayed using peripheral blood mononuclear cells (PBMC) as target cells, as previously described www.europrise.org/neutnet_sops.html; SOP3B. It is important to note that the PBMC-based assay differs from the TZMbl assay in target cell, use of virus isolates and readout of virus replication after seven days using p24 antigen quantification.

### Peptide competition neutralization assays

Peptide inhibition of IgG neutralization was measured using a modified pseudovirus neutralization assay [33] in which the purified IgGs were pre-incubated for 30 minutes with peptide dissolved in DMSO at a final concentration of 16 µg/ml prior to addition of the pseudovirus. The cyclic HIV-1 MN V3 peptide (CTRPNYNKRKRIHIGPGRAFYTTKNIIGTIRQAHC, EVA7019), the linear GPGR HIV-1 SF2 clade B V3 peptide (TRKSIYIGPGRAFHTT, ARP797), the linear GPGQ HIV-1 consensus clade A peptide (KSVHIGPGQAFYAT, ARP7012.1) and the scrambled control (ARP7099) were obtained from the EVA Centre for AIDS Reagents, NIBSC, UK.

### Statistical Analysis

Figures as well as statistical analyses were prepared using GraphPad Prism software Version 5.03 (GraphPad Software, La Jolla, CA). For comparisons of ELISA titers and neutralization responses two tailed Mann-Whitney tests were performed unless otherwise stated.

## Results

### Expression and purification of Env proteins

**Figure 1:**
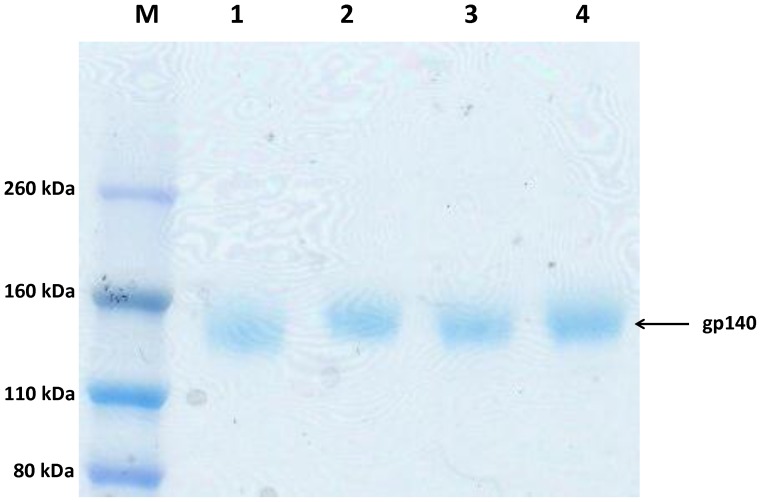
Analysis of purified gp140 proteins. Reduced SDS-PAGE gel (10–12%) analysis of four gp140 proteins (lane 1 to 4) after purification and staining with Coomassie Blue. Lane 1 ITM1_anc; lane 2 ITM1_4; lane 3 ACS19642; lane 4 ACS19554. Proteins were run next to a molecular weight marker (Novex Sharp Pre-stained protein standard, Invitrogen). Proteins were typically >95% pure prior to immunisation.

To improve the presentation of Env glycoproteins in their trimeric forms all Envs were codon optimized and the protease cleavage site REKR was replaced by SEKS to improve stability of the trimers. The *env* gene including the gp120 region and ending with the ELDKWAS, just prior to the transmembrane region of gp41, was cloned into an expression vector (pLEXm) downstream of a tissue plasminogen activator (tpa) leader sequence, and with an N-terminal His tag to allow purification of the protein. After the *in vitro* bulk transient expression and purification the proteins used for immunization were typically >95% pure as analysed by SDS-PAGE under reducing conditions and Coomassie staining (Figure 1). The sequences of all Envs were deposited at GenBank (accession numbers AF062521, GU455427, GU455458, FM165626, KF061033, KF061031, and KF061032).

### Comparisons of monomeric gp120 and trimeric gp140 Env immunizations

In order to investigate whether trimeric gp140 and monomeric gp120 differ with regard to eliciting NAb responses, four rabbits were immunized with either gp120 or gp140 proteins from a reference subtype B strain, Bx08 [34], [35] in a pilot experiment. Four immunizations at weeks 0, 2, 4 and 8 were administered using 20 µg protein per dose in the presence of CAF01 adjuvant. Anti-Env binding titers, as measured by ELISA, were similar and the mean titers (MT) for the monomeric gp120 Bx08 and trimeric gp140 Bx08 four weeks after the last immunization (week 12) were 6.6×10^4^ and 2.4×10^4^ respectively.

To avoid non-specific reactivity of rabbit sera in the TZMbl assay, IgG was purified and used at a maximum concentration of 250 µg/ml, corresponding to ∼1∶50 serum dilution. At this concentration IgG from pre-immune sera resulted in low (10–20%) background neutralization in the TZMbl assay (data not shown).

**Figure 2:**
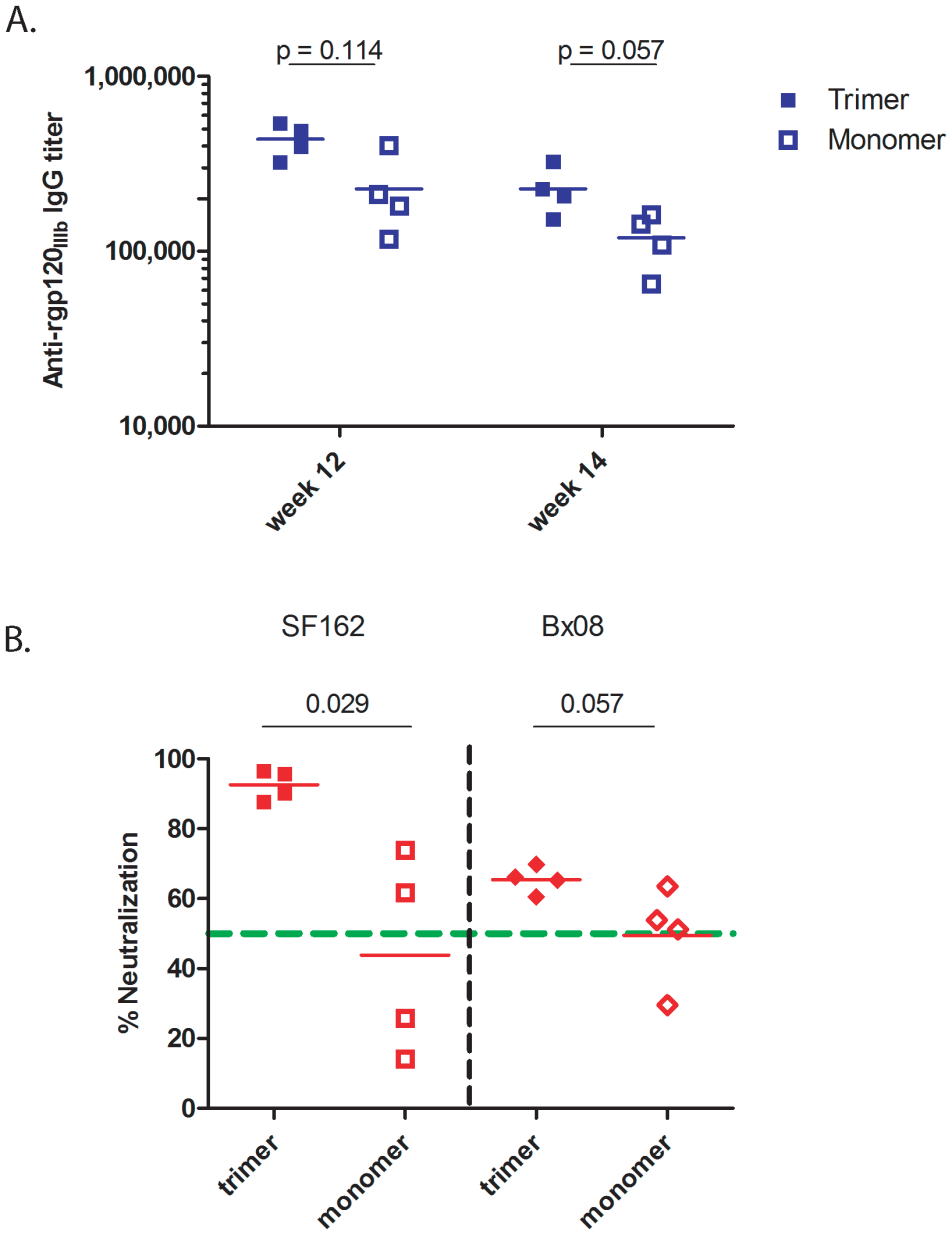
Comparison of trimeric gp140 versus monomeric gp120. (A) End-point binding titers of rabbits immunized with either trimeric gp140 (filled squares) or monomeric gp120 (open squares) of UG_A HIV-1 Env. Each dot represents one rabbit. Horizontal lines indicate mean titers. Statistical analysis was done using Mann-Whitney test. (B) Neutralization data using IgG (at a final concentration of 250 µg/ml) isolated from week 12 sera of rabbits immunized, in the presence of CAF01, with either trimeric gp140 or monomeric gp120 of UG_A. Neutralization of two clade B viruses, SF162 (left, squares) and Bx08 (right, diamonds) is depicted. Each dot represents one rabbit. Horizontal lines indicate the mean percent neutralization. Statistical analysis was done using Mann-Whitney test.

However, when using 250 µg/ml IgG from rabbits of both groups no neutralization of SF162 pseudovirus was observed (data not shown). Since no neutralizing activity and rather modest binding antibody titers were elicited in this pilot experiment the antigen doses were increased from 20 µg to 100 µg in the next experiments. Here monomeric gp120 and trimeric gp140 of the subtype A, UG_A Env were compared following the same immunization schedule. In this case the mean anti-Env binding titers in trimeric gp140 immunized rabbits were slightly higher (4.4×10^5^ and 2.3×10^5^) as compared to the monomeric gp120 immunized rabbits (and 2.3×10^5^ and 1.2×10^5^) at week 12 and 14 respectively (p = 0.114 and 0.057) (Figure 2a). Neutralization responses against the SF162 pseudovirus were significantly higher (p = 0.029) in the rabbits immunized with trimers as compared to monomers, both at week 12 (Figure 2b) and 14 (data not shown). There was also a difference, although not significant, in neutralizing activity against the Bx08 pseudovirus (Figure 2b; Table S2). Taken together these results suggest that the trimeric gp140 Env immunogen is superior to monomeric gp120 in eliciting NAb.

### The need of an adjuvant to elicit potent NAbs by gp140 trimer vaccination

**Figure 3:**
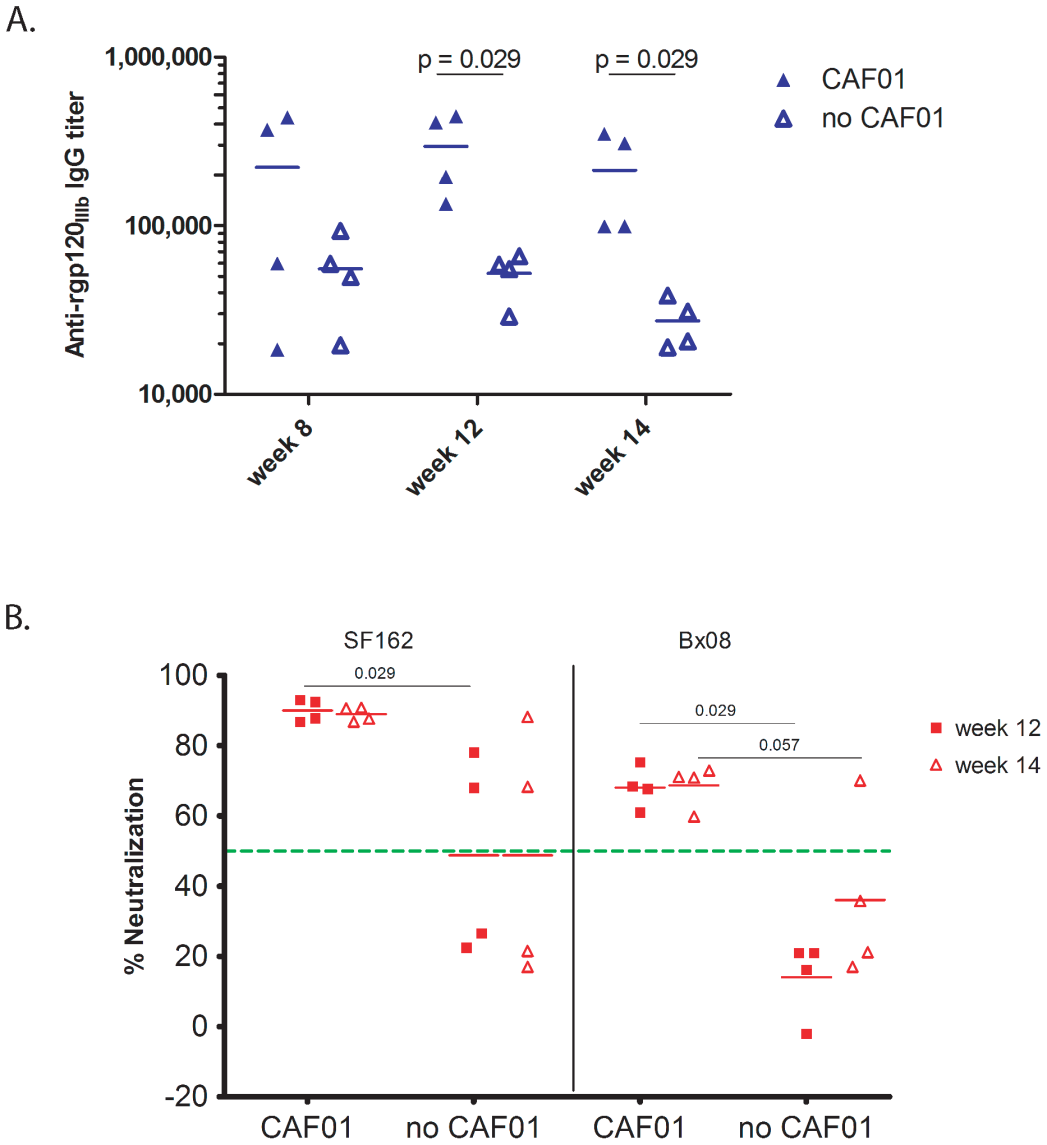
Immunization in the presence or absence of CAF01. (A) End-point binding titer of rabbits immunized with ITM1_4, in the presence (filled triangles) or absence (open triangles) of CAF01 respectively. Results are given for weeks 8, 12 and 14. Each dot represents one rabbit. Horizontal lines indicate mean titers. Statistical analysis was done using Mann-Whitney test. (B) Neutralization using IgG (at a final concentration of 250 µg/ml) isolated from week 12 (filled squares) and 14 (open triangles) sera of rabbits immunized with ITM1_4 in the presence or absence of CAF01. Two clade B viruses, SF162 (left) and Bx08 (right) were used. Horizontal lines indicate the mean percent neutralization. The dotted (green) line represents 50% neutralization. Statistical analysis was done using Mann-Whitney test.

In order to ascertain whether an adjuvant was important for eliciting NAbs in rabbits, immunization with ITM1_4 gp140 trimer was performed both in the presence and absence of the CAF01 adjuvant. Four weeks after the last immunization (at week 12) titers of binding anti-Env antibodies were found to be significantly higher (p = 0.029) in the presence of CAF01 as compared to the administration of the gp140 trimer only (Figure 3a). Also, neutralizing IgG responses against both SF162 and Bx08 were significantly higher (p = 0.029) when CAF01 was included in the immunizations (Figure 3b). Thus, these findings demonstrate that an adjuvant triggering high titer Env binding antibodies is needed for eliciting potent NAbs in rabbits immunized with gp140 trimer.

### Comparison of immunogenicity and induction of Tier 1 NAbs by selected gp140 trimers

**Figure 4:**
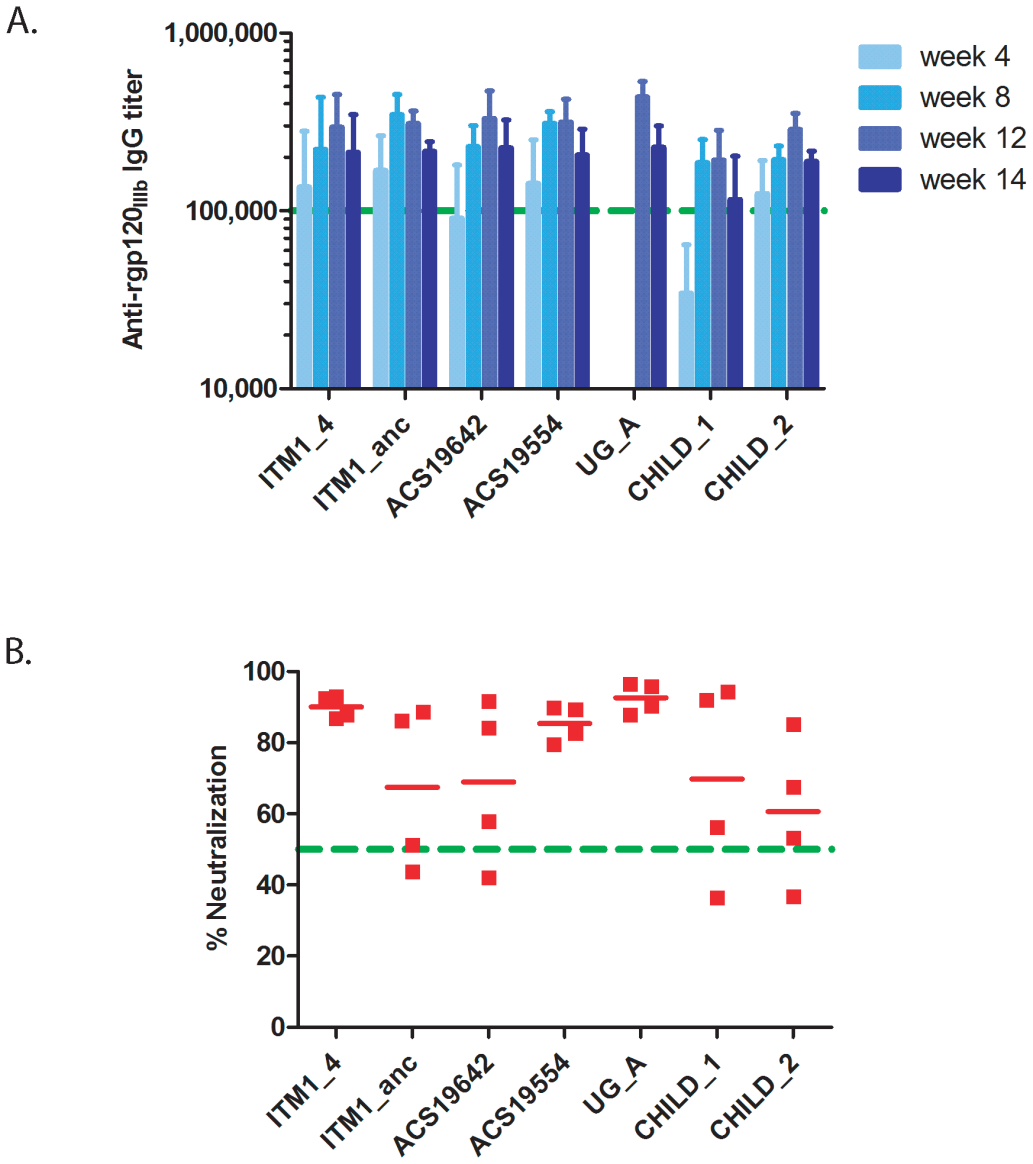
Comparison of Env immunogens. (A) Kinetics and comparison of end-point binding titers of all rabbits immunized with Env gp140 trimers (100 µg/dose) in the presence of CAF01. The mean of four rabbits/group with standard deviation is given for weeks 4, 8, 12 and 14. For UG_A the data for week 4 and 8 are lacking. The green dotted line represents an end-point titer of 10^5^. No statistical differences between the various trimeric gp140 Envs were observed (p = 0.18 and 0.61 at week 12 and 14 respectively; Kruskal-Wallis). (B) Neutralization of SF162 virus using IgG (at a final concentration of 250 µg/ml) isolated from week 12 sera of rabbits immunized with trimeric gp140, all in the presence of CAF01. Each dot represents one rabbit. Horizontal lines indicate mean percent neutralization. The dotted (green) line represents 50% neutralization. Overall, no statistical differences were found between the trimeric gp140 Envs (p = 0.092, Kruskal-Wallis).

In the next step we compared side-by-side the seven selected Env gp140 trimers (Table 1) for their immunogenicity and capacity to elicit NAb responses in the presence of adjuvant. In all groups of rabbits we observed an increase of binding titers that peaked at 8 or 12 weeks (i.e. just before or 4 weeks after the last immunization) (Figure 4a). When analyzed at week 12 and 14 the mean anti-Env gp120 binding titers were similar between the groups of rabbits immunized with the different gp140s (p = 0.18 and 0.61, respectively) and ranged between 1.9×10^5^ (CHILD_1) – 4.4×10^5^ (UG_A) and 1.2×10^5^ (CHILD_1) – 2.3×10^5^ (UG_A), respectively. Furthermore, the overall mean binding antibody titer dropped from 3.1×10^5^ at week 12 (i.e. 4 weeks after the last vaccination) to 2×10^5^ at week 14 (Table S1, Figure 4a).

**Table 1:** List of selected immunogens.

Immunogen	information patient	time post infection	subtype	gp140		Reference
				length^a^	PNGS^b^	
Bx08	primary, CCR5 tropic virus isolated early after infection	8 months	B	666	31	Aids Res Hum Retroviruses 1997; 13:19–27
ITM1_4	Long term survivor infected at birth; Rwanda	11 years	A	671	29	J. Virol. 2007; 81:6548-62
ITM1_anc	Predicted ancestral sequence (based on 11 years follow-up env sequences)		A	692	32	
ACS19642	Long term non progressor, sensitive to autologous neutralization; The Netherlands	29 months	B	669	25	J. Virol. 2010; 84:3576-85
ACS19554	Progressor, sensitive to autologous neutralization; The Netherlands	47 months	B	679	32	J. Virol. 2010; 84:3576-85
94UG018 (UG_A)	Asymptomatic pregnant woman; Uganda	unknown	A	694	33	J. Virol. 1995; 69:7971-81
306-9 (CHILD_1)	Delayed progressor infected at birth; Italy	9 months	B	678	30	Aids Res Hum Retroviruses 2007; 23:1531-40
136-3 (CHILD_2)	Slow progressor infected at birth; Italy	3 months	B	665	25	Nat Medicine 1997; 11:1259-65

Bx08 was used as benchmark in a pilot experiment.

a length: number of amino acids.

b PNGS: number of potential N-linked glycosilation sites as identified using N-glycosite at the HIV database website (http://www.hiv.lanl.gov/content/sequence/GLYCOSITE/glycosite.html).

In order to compare the neutralization activity of Abs induced by the various Envs, IgG purified from sera collected at week 12 was tested in the TZMbl assay. It was evident that certain immunogens induced less variable antibody responses (Figure 4b), although the group averages were not significantly different (p = 0.092). Nevertheless, in the groups immunized with the ITM1_4, ACS19554 and UG_A trimers, all rabbits had IgG that showed > 80% neutralization of SF162 at the highest IgG concentration (250 µg/ml) used (mean neutralization 90%, 85% and 93% for ITM1_4, ACS19554 and UG_A, respectively). In contrast, despite the fact that all trimers used elicited similar anti-Env binding titres, the neutralizing activity of IgG in those groups immunized with gp140s of ITM_anc, ACS19642, CHILD_1 and CHILD_2 was less consistent and was rather low in at least two out of the four immunized rabbits (Figure 4b).

**Figure 5:**
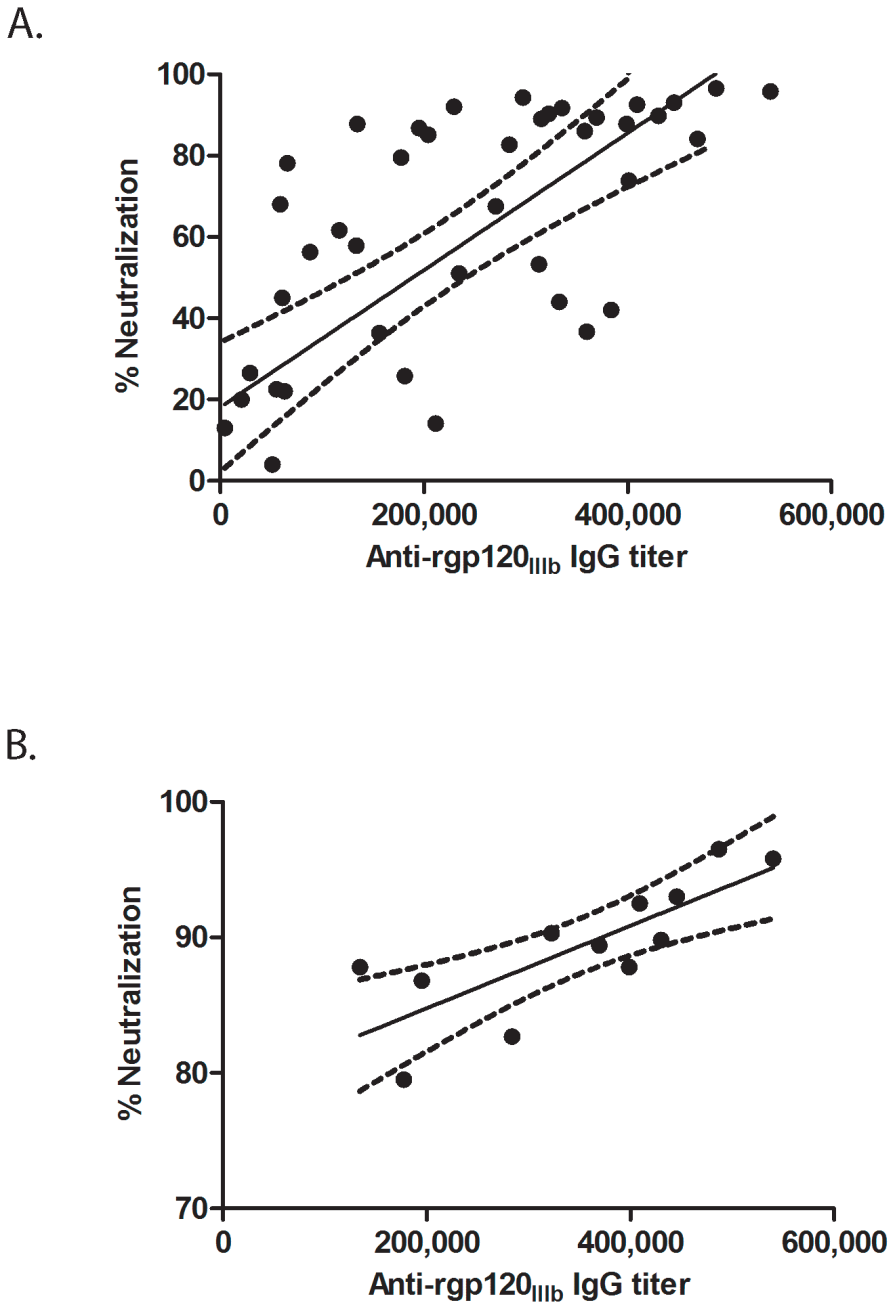
Correlation between end-point binding titers and neutralization responses against SF162. (A) Correlation using week 12 sera (ELISA) or IgG (neutralization responses at a final concentration of 250 µg/ml) of all groups, including the monomers (44 data points). Spearman correlation r = 0.7243; p <0.0001. (B) Correlation using week 12 sera (ELISA) or IgG (neutralization responses at a final concentration of 250 µg/ml). Only data from rabbits immunized with trimers ITM1_4, UG_A and ACS19554, giving the highest neutralization responses (12 data points) were used. Spearman correlation r = 0.8616; p = 0.0003.

These results prompted us to look in more detail for correlations between binding Ab titers and NAb responses 4 weeks after the last immunization (week 12). When all results were considered, regardless immunogen and dose, a significant correlation between neutralizing activity and end-point binding Ab titer was found (r = 0.7243; p <0.0001) (Figure 5a). We also noted that the correlation was stronger (r = 0.8616; p = 0.0003) when only the groups (ITM1_4, ACS19554 and UG_A trimers) with the highest NAb responses were considered (Figure 5b).

In addition, a threshold of 10^5^ binding Ab titer for detection of neutralizing IgG activity was supported, both in the TZMbl assay as well as the PBMC-based assay where IgG from groups immunized with gp140 trimers of Bx08, ITM1_4, ITM1_anc, ACS19554 and ACS19642 were tested in parallel against SF162 (Figure S1). It is important to note that in the experiment with Bx08 only 20 µg protein per vaccination was given. In addition, similar to results obtained in the TZMbl assay, the PBMC-based assay revealed a direct correlation between neutralizing activity and titers of gp120 binding antibodies, r = 0.6616; p = 0.0015 (Figure S1). Taken together, these results suggest that potent NAb responses in rabbits depend on the induction of high-titer binding Abs, however, the presence of high-titer binding Abs does not always translate into potent NAbs.

#### Kinetics and breadth of NAb responses

**Figure 6:**
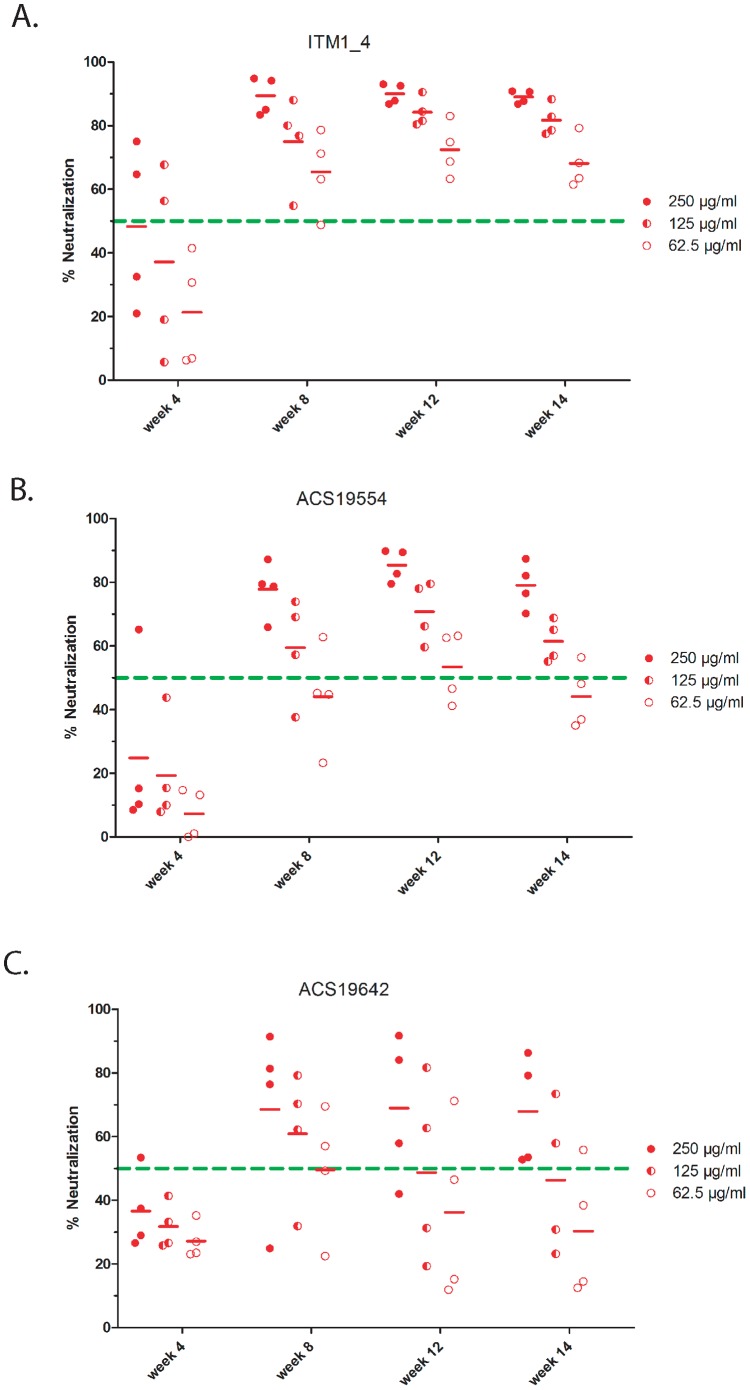
Kinetics of neutralizing responses against SF162 pseudovirus. Kinetics using three 2-fold dilutions of IgG isolated from weeks 4, 8, 12 and 14 sera of rabbits immunized with trimer ITM1_4 (Figure 6a), ACS19554 (Figure 6b) and ACS19642 (Figure 6c). Horizontal lines indicate the mean percent neutralization. Final IgG concentrations were 250 µg/ml (filled dots), 125 µg/ml (semi filled dots) and 62.5 µg/ml (open circles) respectively. The dotted (green) line represents 50% neutralization.

To analyze the kinetics of the antibody responses to immunization we more closely compared binding Abs and NAb in two groups with a strong response (ITM1_4 and ACS19554) and in one group with a weaker response (ACS19642). To this end, three concentrations of purified IgG were tested at four time points (Figure 6a-c). Clearly, as early as two weeks after the 2^nd^ immunization (week 4) NAb were raised in some of the rabbits, but there was heterogeneity in all groups. However, from week 8 (i.e. 4 weeks after the 3^rd^ vaccination) the “high” and “low” responding groups differentiated from each other in that near optimal and homogenous neutralization levels were reached in the rabbits of groups immunized with ITM1_4 and ACS19554, whereas the response remained heterogeneous and lower in the group immunized with ACS19642. At week 12 (4 weeks after the last immunization) the neutralization capacity slightly increased in groups immunized with ITM1_4 and ACS19554 while in the group immunized with ACS19642 there was no improvement of the neutralization capacity. Six weeks after the last immunization (week 14) NAb titers tended to decrease.

The breadth of NAb responses was further investigated against pseudoviruses belonging to Tier 1 subtype B (Bx08), C (MW965.26) and CRF02_AG (DJ263.8) in the TZMbl assay (Table 2). Of note, ITM1_4 trimers induced the broadest response, followed by UG_A. All gp140s obtained from early isolates, including the deduced ancestral sequence (ITM1_anc), induced a moderately broad or no response.

**Table 2:** Mean percent neutralization of immunogens as tested against Tier 1 viruses.

	immunogen		CHILD_2	CHILD_1	ITM1_anc	ACS19642	ACS19554	ITM1-4	UG_A
	*time post infection*		*3 months*	*9 months*	*ancestral*	*29 months*	*47 months*	*11 years*	*unknown*
			mean % NT/group a						
CLADE	VIRUS	TIER	week 12						
B	SF162	1A	61	70	67	69	85^b^	90	93
B	Bx08	1A	25	40	54	42	65	68	65
C	MW965.26	1A	ND^c^	ND	ND	86	93	96	95
CRF02_AG	DJ263.8	1B	ND	ND	ND	46	34	67	38
	*Mean % NT*		43	55	61	61	69	80	73
			week 14						
B	SF162	1A	46	64	64	68	79	89	79
B	Bx08	1A	22	33	57	43	61	69	52
C	MW965.26	1A	ND	ND	ND	77	91	87	89
CRF02_AG	DJ263.8	1B	ND	ND	ND	52	20	63	55
	*Mean % NT*		34	48	60	58	61	76	69

a Values represent the mean percent neutralization using IgG (250 µg/ml) isolated from week 12 and 14 sera.

b numbers in bold are ≥ 80% neutralization.

c not determined.

Based on these results, purified IgG from sera of rabbits immunized with the most promising trimers ITM1_4, UG_A and ACS19554 were further tested in the TZMbl assay against Tier 2 pseudoviruses of clade A (92RW009), B (QH0692.42) and C (DU174.15, ZM109F.PB4). However, no neutralization activity was detected (data not shown).

#### Peptide inhibition experiments

In order to examine the contribution of V3 directed antibodies to the observed neutralizing IgG activity, we used week 14 IgGs from individual rabbits immunized with ITM1_4 and UG_A, showing the strongest and broadest neutralization activity.

**Figure 7:**
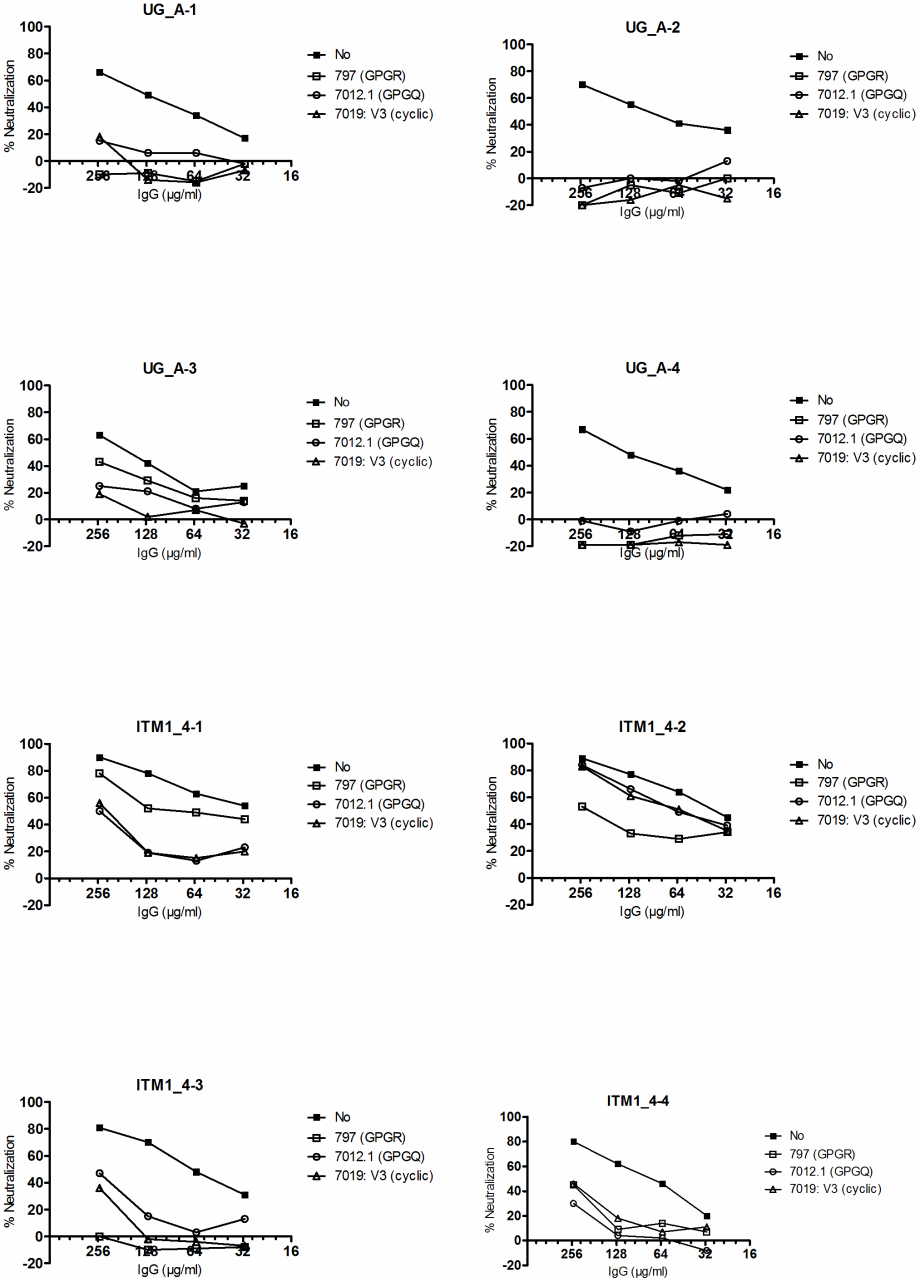
Peptide inhibition experiments. Four serial 2-fold dilutions of IgG purified from rabbits immunized with ITM1_4 (ITM1_4-1; -2; -3 and -4) and UG_A (UG_A-1; -2; -3 and -4) were used in a modified pseudovirus neutralization assay using SF162. Two linear peptides 797 and 7012.1, recognizing the V3-crown motif of clade B (GPGR) and clade A (GPGQ) respectively and 1 cyclic peptide 7019 recognizing GPGR (clade B) were used at a final concentration of 16 µg/ml. Each graph represents a single rabbit.

The neutralization of SF162 was assessed in the presence of peptides that comprised the entire V3 region of MN or shorter linear peptides that were localized centrally on the V3-crown motifs of clade B or clade A. The neutralization by IgG of 3 out of 4 UG_A immunized rabbits was essentially abrogated by any of the 3 peptides used (Figure 7). In the fourth rabbit (UG_A-3) the cyclic subtype B peptide also strongly inhibited neutralization, but inhibition by the linear peptides, especially the subtype B variant, was weaker. Results in the ITM1_4 immunized animals were different and much more variable: the inhibition by the linear subtype B peptide was complete in one rabbit ITM1_4-3, intermediate in two rabbits ITM1_4-2 and ITM1_4-4 and weak in rabbit ITM1_4-1. The inhibition pattern by the cyclic subtype B and the linear subtype A peptides was remarkably similar in each individual rabbit of this group, but differed between individuals: intermediate in ITM1_4-1, ITM1_4-3 and ITM1_4-4 and very weak in ITM1_4-2. No inhibition of neutralization was observed using the scrambled peptide (ARP7099) (data not shown).

Overall, these data suggest that V3-specific neutralizing IgG was present in all analyzed rabbits, that this was more important in UG_A immunized as compared to ITM1_4 immunized ones, and that significant individual variability with regard to the extent and fine specificity was noted.

## Discussion

In this study we investigated whether a rational selection of Envs could guide the choice of an immunogen, able to elicit broadly neutralizing antibody responses in a rabbit model. To this end, we selected patients with broadly neutralizing antibodies and amplified the *env* gene of their viruses, obtained either shortly after infection [23], [26] or during the chronic phase [20], [21], [22]. From our results it appears that the Envs from the chronically infected patients (ITM1_4, UG_A and ACS19554) were able to elicit more potent NAbs as compared to the early Env sequences obtained within 2.5 year post-infection. In line with these results, when using an artificially constructed ancestral Env sequence (ITM1_anc), less potent NAb responses were generated.

Comparing the monomeric gp120 with the trimeric gp140 form of the same precursor Env sequence (UG_A) demonstrated the latter to be superior in elicitation of both binding titers and neutralizing responses. This is in agreement with the findings of others [36], [37], [38], [39], [40], [41].

Interestingly, for the three immunogens (ITM1_4, UG_A and ACS19554) that yielded the best NAb responses, a strong correlation between end-point binding titers and neutralization responses against SF162 was found. The latter finding might point to the importance of a minimal threshold that should be reached after immunization before NAbs can be detected, but around this threshold the quality of the immunogen becomes the most important factor in determining the quality of the induced NAb responses.

From our experiments it could also be concluded that both the amount of protein used and the presence of an adjuvant are of major importance in inducing antibody responses. In contrast to Lai et al [42] we found that binding titers substantially differed with the amount of protein administered and high titers were only obtained when using 100 µg trimer per immunization.

Neutralization was cross clade (B, CRF02 and C) but restricted to Tier 1 viruses which is in 

agreement with a previous vaccine study performed in rabbits [36] where trimer vaccination induced Tier 1 neutralizing responses in the TZMbl assay. Similar with this study we also found (unpublished data) that in rabbits DNA prime - protein boost did not result in an increased magnitude or breadth of NAb responses as compared to protein (trimer) vaccination alone. Two studies conducted in guinea pigs showed that trimer vaccination induced Tier 1 as well as Tier 2 virus neutralization in the A3R5 [40] or TZMbl assay [43], suggesting that, although immunogens are not comparable between the studies, it is easier to obtain Tier 2 virus neutralization in guinea pigs than in rabbits.

Peptide inhibition experiments suggested that a significant proportion of the neutralizing response was targeted against the V3 portion of Env, both in UG_A and ITM1_4 immunized rabbits. This observation supports the view that the variable loops of the Env, and in particular V3, are indeed important for vaccine design. In fact, despite their sequence diversity, they do have conserved immunologic elements capable of eliciting highly cross-reactive antibodies [44].

The rabbit model is widely used in HIV-1 vaccine development due to its relative size which yields large amounts of blood for testing, yet rabbits are small enough for relatively inexpensive handling on a larger scale. More importantly, the length of the antibody heavy chain complementary-determining region three (CDR3) is longer and closer to the CDR3 length in humans as compared to other small animal models e.g. the mouse. Long and flexible CDR3 have been claimed to be important for the good neutralizing capacity of some human antibodies [45], [46]. Although slightly different results may have been obtained using a different small animal model like guinea pigs or a different immunization schedule allowing for antibody maturation, the use of the same animal model and immunization schedule did allow for direct comparison of the differently selected immunogens. Additional optimizations of delivery may be needed for *in vivo* evaluations of protection.

In conclusion, our results indicate that the strategy of reverse immunology based on select Env sequences is promising and that the rabbit is a valuable model for comparison of selected immunogens to be used in HIV vaccine studies. Clearly, however, additional optimizations of the proposed immunogens may be needed before *in vivo* evaluations of protection is attempted.

## Supporting Information

Figure S1 **PBMC neutralization data and correlation with end-point binding titers**. (A) Neutralization of SF162 virus using IgG (at a final concentration of 125 µg/ml) isolated from week 14 sera of rabbits immunized with 100 µg/dose trimeric gp140 in the presence of CAF01. Note that using Bx08 only 20 µg/dose was used. Each dot represents one rabbit. Horizontal lines indicate mean percent neutralization. A significant difference (p = 0.028, Kruskal-Wallis) was observed between rabbits immunized with 20 µg Bx08 trimeric gp140 and rabbits immunized with 100 µg trimeric gp140. (B) Correlation between end-point binding titers and neutralization responses against SF162 using sera (ELISA) and IgG (neutralization responses at a final concentration of 125 µg/ml) from week 14. Data from rabbits immunized with trimeric Bx08 (red triangles), ITM1_4, ITM1_anc, ACS19642 and ACS19554 (black dots) were used. Spearman correlation r = 0.6632; p = 0.0014.
(TIFF)

Table S1 **Mean end-point binding titers and neutralizing antibody responses.** Results for all groups receiving trimeric gp140 and the group receiving monomeric gp120 of UG_A is given. The TZMbl neutralization data (SF162) were obtained using IgG at 250 µg/ml. MT: overall mean titer. SEM: standard error of the mean.
(XLSX)

Table S2 **Neutralizing antibody responses against Tier 1 pseudoviruses.** Percentage neutralization of individual rabbits as tested against Tier 1 viruses belonging to subtype B (SF162 and Bx08), subtype C (MW965.26) and CRF02_AG (DJ263.8) are given. The data is color coded: Green represents between 50 and 69% neutralization, yellow represents between 70 and 84% neutralization and red represents between 85 and 100% neutralization. ND: not determined.
(XLSX)
