# Supplementary figures and images for: Selected HIV-1 Env Trimeric Formulations Act as Potent Immunogens in a Rabbit Vaccination Model
Source: PLoS One. 2013 Sep 2;8(9):e74552. doi: 10.1371/journal.pone.0074552 (PMC3759472; doi:10.1371/journal.pone.0074552)

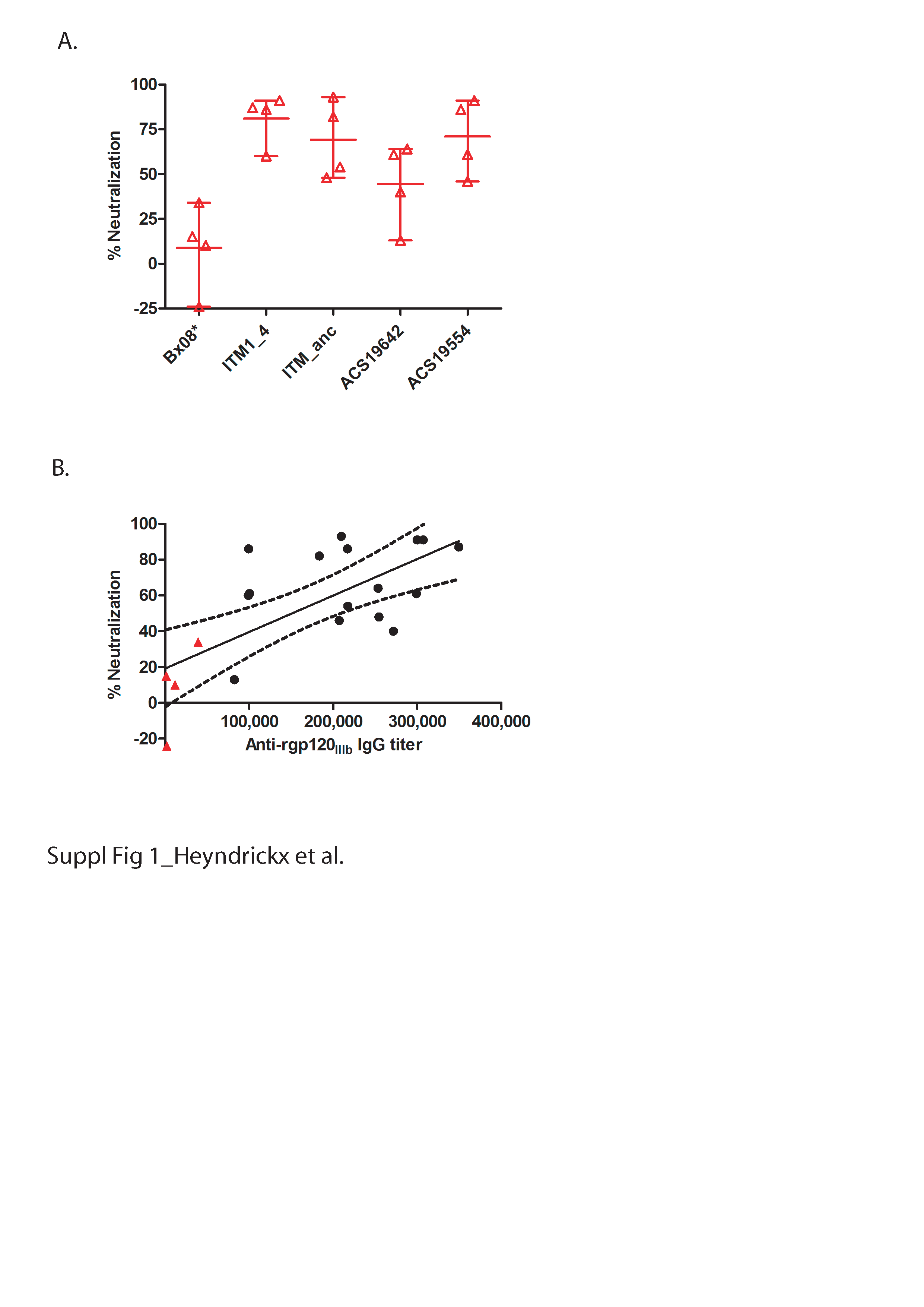

Supplement: Figure S1 — PBMC neutralization data and correlation with end-point binding titers. (A) Neutralization of SF162 virus using IgG (at a final concentration of 125 µg/ml) isolated from week 14 sera of rabbits immunized with 100 µg/dose trimeric gp140 in the presence of CAF01. Note that using Bx08 only 20 µg/dose was used. Each dot represents one rabbit. Horizontal lines indicate mean percent neutralization. A significant difference (p = 0.028, Kruskal-Wallis) was observed between rabbits immunized with 20 µg Bx08 trimeric gp140 and rabbits immunized with 100 µg trimeric gp140. (B) Correlation between end-point binding titers and neutralization responses against SF162 using sera (ELISA) and IgG (neutralization responses at a final concentration of 125 µg/ml) from week 14. Data from rabbits immunized with trimeric Bx08 (red triangles), ITM1_4, ITM1_anc, ACS19642 and ACS19554 (black dots) were used. Spearman correlation r = 0.6632; p = 0.0014. (TIFF) [file pone.0074552.s001.tiff]
